# Nutrition Intervention of Groceries for Black Residents of Boston to Stop Hypertension (GoFresh) Among Adults With or Without Treated Hypertension Trial: Rationale, Design, and Evidence to Promote Implementation

**DOI:** 10.21203/rs.3.rs-6222158/v1

**Published:** 2025-07-07

**Authors:** Kayla M. Ferro, Reva Seager, Kathy McManus, Kristen M. Kraemer, Ruth-Alma Turkson-Ocran, Jackie Michetti, Sofia Allison, Stephanie L. Fitzpatrick, Stephen P. Juraschek

**Affiliations:** Beth Israel Deaconess Medical Center, Division of General Medicine, Section for Research, Boston, MA; Beth Israel Deaconess Medical Center, Division of General Medicine, Section for Research, Boston, MA; Harvard Medical School, Boston, MA; Department of Nutrition, Brigham and Women’s Hospital; Beth Israel Deaconess Medical Center, Division of General Medicine, Section for Research, Boston, MA; Harvard Medical School, Boston, MA; Beth Israel Deaconess Medical Center, Division of General Medicine, Section for Research, Boston, MA; Harvard Medical School, Boston, MA; Beth Israel Deaconess Medical Center, Division of General Medicine, Section for Research, Boston, MA; Beth Israel Deaconess Medical Center, Division of General Medicine, Section for Research, Boston, MA; Kaiser Permanente Center for Health Research, Portland, Oregon, USA; Beth Israel Deaconess Medical Center, Division of General Medicine, Section for Research, Boston, MA; Harvard Medical School, Boston, MA

**Keywords:** hypertension, medically tailored groceries, nutrition, DASH diet, dietitian

## Abstract

**Background:**

The Dietary Approaches to Stop Hypertension (DASH) Eating Plan is proven to lower blood pressure; however, the original DASH diet involved a set menu of meals prepared in a metabolic kitchen. There is little evidence mapping this dietary pattern to real-world groceries, tailored to a range of cultural preferences and dietary practices.

**Methods:**

The GoFresh Trial, a parallel-arm randomized, controlled trial, is studying the impact of DASH-patterned, home-delivered groceries on the blood pressure of Black adults living in communities with reduced access to grocery stores. Participants were able to choose DASH-patterned groceries according to their preferences for themselves and up to five family members from local supermarkets. A dietitian assisted participants with grocery selection to ensure that groceries followed a DASH pattern and met potassium/sodium ratio of >2.2 with kilocalories from saturated fat ≤7%. In addition, dietitians provided weekly educational modules on sustainably adopting DASH. Two conceptual frameworks were designed to address five domains related to diet adoption: accessibility and cost, food preparation, social influences, individual beliefs, and cultural adaptations. To support meal preparation, a recipe book and 24 demonstration videos were created in collaboration with Boston chefs to highlight heritage diets like African and Afro-Caribbean.

**Results:**

Compliance assessments include 24-hour urine paired with 24-hour nutrition recalls, seated blood pressure, and surveys collecting information on food preparation and shopping habits.

**Conclusion:**

Findings from this study will inform policy related to healthy food access and provide real-world examples of how DASH might be adapted in a real-world context now and in years to come.

## Introduction/Background

Hypertension is one of the most important modifiable risk factors for cardiovascular disease.^1^ Black persons are disproportionately impacted by hypertension and there is strong evidence that diet is a primary mediator of disparities in hypertension among Black adults.^2^ Prior work through the Dietary Approaches to Stop Hypertension (DASH) and DASH-Sodium trials demonstrated meaningful improvements in cardiovascular (CVD) risk factors through consuming a low sodium, DASH diet with greater effects among Black adults.^3,4^ However, translation of these dietary patterns has been challenging for several reasons. First, the original DASH diets were prepared in a metabolic kitchen, and while these meals could theoretically be recreated by consumers in the U.S., they were not commercially available in real-world settings. Moreover, the original DASH meals followed a fixed meal plan based on typical American dietary patterns in the 1990s. This may not be acceptable across multicultural populations or a range of geographic locations throughout the U.S. or internationally. Finally, nutrition insecurity resulting from poor access to healthy foods is a prevalent, significant, and growing barrier to the adoption of healthy eating and the realization of its health benefits.^5^

There is increasing recognition of the importance of home-delivered groceries to promote healthy dietary patterns in the U.S. and throughout the world.^6–8^ Unlike medically-tailored meals, medically-tailored groceries have tremendous potential for adaptation and customization to an extensive range of distinct cultural heritages that can enable translation of DASH principles across cultures and geographic settings. Prior work has demonstrated the importance of cultural adaptation to achieve long-term adherence.^9^ Moreover, a number of grocery intervention studies demonstrated improvements in healthy eating by supporting participants’ choice of food.^10–12^ However, these studies have fallen short of demonstrating direct benefits on blood pressure and related CVD risk factors (e.g., low density lipoprotein cholesterol or markers of glycemia). This may be in part due to the insufficient amount of food replacement provided through prior work or missing key food groups that were important for achieving the full benefits of the DASH diet.

Through the GoFresh and GoFreshRx trials, this dietitian-led nutrition intervention aimed to implement a DASH framework for grocery selection in a personalized, person-centered manner that addressed barriers related to accessing DASH groceries and potential gaps in knowledge and skills related to preparing DASH meals. The goal of this manuscript is to describe the design of the intervention with implementation case examples and serve as a guide for practitioners seeking to translate this intervention into real-world clinical encounters.

## Design

### Parent study design and population

Details of the GoFresh design were published previously.^13^ In brief, the GoFresh trials each enrolled up to 176 participants who self-identified as Black or African American, were 18-years or older, and lived in areas characterized by a low concentration of grocery stores in the Boston area. The only difference between GoFresh and GoFreshRx was hypertension treatment status: GoFresh enrolled participants without hypertension medications, while GoFreshRx focused on adults on stable hypertension treatment. All participants were required to have a systolic blood pressure of 120 to <150 mm Hg and a diastolic blood pressure <100 mm Hg based on the average across three screening visits. Participants were excluded if they were taking medications for diabetes, reported severely limited dietary preferences, allergies, or malabsorption, among others. Examples of dietary exclusions included vegan diets, gluten-free due to Celiac Disease or gluten allergy, or unwilling to eat one or more of the seven DASH food groups. Adjustments were made to include vegetarians, as long as they ate nuts/seeds/legumes and dairy (or non-dairy) products in order to achieve a DASH dietary pattern. More details on how the intervention was adapted for vegetarians may be found in the vegetarian case example (Case 1, **Supplement Material SM1**). Non-dairy products were provided for folks with lactose intolerance (Case 2, **Supplement Material SM1**).

### Assignments

The intervention was divided into two arms; A) self-directed shopping and B) dietitian-assisted DASH grocery delivery. The self-directed shopping group (the reference group) received an introduction to the DASH plan and an unrestricted stipend of $500/month (at 4-, 8-, and 12-weeks post randomization). Before each payment, they completed a virtual check-in with their assigned dietitian.

Participants randomized to the active intervention, i.e., dietitian-assisted, home-delivered DASH groceries, partook in weekly grocery order calls (GOs) with the dietitian plus a home-delivered grocery order following the DASH Eating Plan. These calls served three purposes: (1) order groceries for the week, (2) assess compliance from the prior week’s order, and (3) allow for education and counseling on fundamentals of the DASH principles. This group was asked to restrict food consumption to the study groceries for the entire 12-week intervention.

### Two Conceptual Frameworks

**Figure 1:**
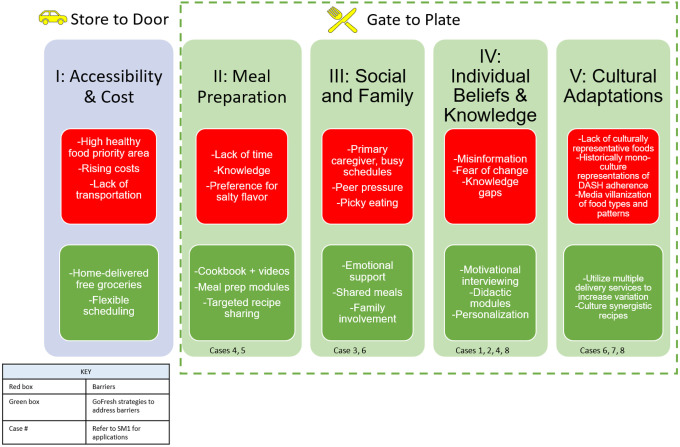
Addressing Barriers to Adopting the DASH Eating Plan

The intervention sought to address multiple domains related to barriers in adopting the DASH Plan through two proposed frameworks, ‘store-to-door’ and ‘gate-to-plate’ (See Figure 1). GoFresh identified five domains as major contributors to adopting a healthy diet: I) accessibility and cost, II) cooking skills and knowledge, III) social and family influences, IV) individual beliefs and knowledge, and V) cultural adaptation. The ‘store-to-door’ framework, similar to a traditional food-is-medicine approach, focused on both (a) selection of foods that met specific nutrient goals in a manner consistent with participant personal preferences as well as (b) increasing access to healthy foods by using online grocery stores to deliver groceries directly to people’s homes. The “gate-to-plate’ framework focuses on barriers related to the acceptance, preparation, and consumption of healthy foods (Domains II-V). It addresses how to support people to actually consume the DASH groceries, a step past simply receiving them at their door. Some may argue that getting the food to people’s home is the easiest step, however, encouraging acceptance of the foods and actual consumption requires more time and effort, preferably with a nutrition professional. Domains II-V can be seen in practice in **Supplement Material SM1**.

### Grocery orders and nutrient information

An order sheet was developed to record, track, analyze, and guide the weekly DASH grocery order. It was divided into seven sections for the seven food groups of DASH; fruit, low-fat dairy, protein, fats/oils/spices, vegetables, grains, nuts/seeds/legumes. Energy needs were calculated using the Mifflin St. Jeor equation.^17^ After the caloric level was determined, the dietitians used an adapted version of the DASH Eating Plan—Number of Food Servings by Calorie Level from NHLBI (Table 1) to calculate the goal number of servings for the seven DASH food groups selecting the closest calorie level.

The table from the NHLBI’s DASH Eating Plan, “Number of Food Servings by Calorie Level” was adapted to approximate the additional kilocalories and servings needed for family members for at least one meal per day (**see Supplement Table ST2**). The quantity of food was not restricted as part of the intervention and could be adjusted if needed based on feedback from the participants during their weekly GO visit.

Aside from the food item name, the order sheet also listed the order unit, servings per unit, serving size, kilocalories, sodium, and potassium per order. These details made it possible to compare grocery orders to nutrient targets and alternative products were suggested to conform with order goals prior to submitting the order.

Orders prioritized potassium/sodium ratio and proportion of kilocalories from saturated fat, while attempting to maintain recommended food group servings of the DASH diet. If a participant requested an item not on the order sheet, it was added to the order if it met the following DASH requirements per serving: less than 300 mg of sodium and less than 5g of saturated fat. The flexibility to add products at any time point also supported cultural concordance to their current eating pattern (**Domain 5**, Figure 1). GoFresh did not send beverages, sweets, salty snacks, or ultra-processed foods. Organic foods were not emphasized though could be sent if requested. Cost was not a factor in grocery orders. Family size was restricted to 6 adults at dinner due to budget constraints, but the cost of the weekly order was not considered.

### Cookbook and chef collaboration

Recipe development was a collaboration between the dietitians and Black chefs in the Greater Boston area to support incorporation of DASH principles into meals. A GoFresh cookbook was provided to all participants randomized to the dietitian-assisted DASH grocery delivery arm at the start of their intervention and given to the self-directed group after completion of their final visit. Chefs prepared a recipe that the dietitian entered into Elizabeth Stewart Hands and Associates (ESHA) Research’s Food Processor^23^ (Beaverton, Oregon) to extract a nutrition label. Recipes were required to meet study nutrients targets for sodium (less than 300 mg per serving) and saturated fat (less than 5 grams per serving

## Applications of the Gate-to-Plate Model

### Lessons Learned Through ‘The Store to Door’ Framework

Online grocery stores with home delivery are becoming increasingly accessible, offering a growing geographic range that now includes areas of Boston where physical grocery stores may be limited or far from residents’ homes. These virtual stores provide an extensive variety of products that may not be available in the person’s neighborhood. This accessibility supports personalized meal planning and allows adults to maintain dietary preferences aligned with their ethnic traditions.

The goal of the home deliveries was to remove barriers related to accessing DASH-patterned groceries like lack of transportation, cost, and local grocery store availability. The groceries were sent directly to the participant’s home or another convenient location with either Amazon Fresh, Whole Foods, or Instacart. The platform was switched based on participant preference, timing needs of delivery, and the store’s food availability. Delivery times were able to accommodate any work schedule with options in the early morning (before 8AM) and late night (after 10PM) depending on the courier. Multiple store options allowed for greater diversity in food selections.

Online grocery shopping has significantly emerged in the last several years^14^ and GoFresh is one of the first to use these services on a large-scale, nutrition intervention^13^. With this novelty, some roadblocks emerged such as limited food selection, store availability, and skill/knowledge of the third-party shopper. One common complaint of online grocery stores from GoFresh participants thus far was the unreliability of their shoppers. Poor quality and incorrect items were sometimes selected or substituted, which are amplified in a nutrition study because of the strict dietary guidelines. In addition, online vendors were not consistent with providing complete, accurate nutrition information. This limitation is also reported elsewhere.^15,16^ Many times, the dietitians needed to refer to the product company’s website for a complete evaluation of the nutrition label. Culture-specific produce and items were not widely available, such as plantains, yuca, chayote, papaya, fresh collard greens, lima beans, ackee, certain legumes like pigeon peas and lima beans, and barley. The locally owned grocery or corner stores that do sell these products are not regularly available for online shopping.

The delivery of the food itself also exposed barriers related to poor packaging, delivery outside the time window, and failure to follow delivery instructions. This presented challenges for families living in multi-family homes or apartment buildings, especially when groceries were not delivered to the correct door. If 25% or more of a food group’s items were missing, a supplemental order was sent with priority placed on high potassium foods.

### Addressing Barriers Through ‘The Gate to Plate’ Framework

#### General Principles

Dietitian counseling was designed to facilitate the ‘gate to plate’ framework by promoting preparation, consumption, and acceptability of groceries. We used motivational interviewing in a person-centered fashion, emphasizing open-ended questions, rolling-with-resistance, reflective listening, and affirmations. Participants had autonomy over weekly grocery selection and meal preparation, enabling adaptation to a wide range of diverse cultural heritages. During the introduction call, dietitians listened to participants’ descriptions of their eating and food preparation customs and worked to mold the DASH plan to their personal pattern.

#### Through Education

In addition to the grocery order and counseling, a 12-week curriculum was developed to aid in adherence and understanding of the DASH plan (**Supplemental Table ST1**). The curriculum was designed to progress from a more informative, teaching style in the first six weeks, to hands on application and strategy building in the latter six weeks. Topics in the first six weeks included principles of the DASH plan, high potassium foods, potassium and sodium’s effect on the body, understanding the nutrition label, and alternatives to salt. It was designed to deliver novel nutrition information to the participants and build their knowledge of healthy eating. The second six weeks focused on practical goal building to foster long-lasting behavior change. For example, topics in the second six weeks included: how to adhere to DASH at social events, make DASH work for the entire family, develop a personalized DASH shopping list, adapt favorite recipes to DASH guidelines, and set goals to help maintain DASH. During week 12, dietitians reviewed a list of resources in the local community where participants can find DASH groceries, including food banks, farmer’s markets, corner stores, grocery stores, and community health centers.

#### Through Domains II-IV: The Gate-to-Plate Framework

##### Domain II: Meal Preparation

Preparing DASH-compliant and flavorful meals can be challenging for some. Low sodium diets can be bland for those who rely on salt to flavor their food. Therefore, GoFresh Dietitians encouraged seasoning without salt and instead highlighted herbs and spices to bring out the natural flavors of food. Low sodium was encouraged not only through education, but also by including salt-free seasonings in grocery orders. Limited time for food preparation can be another major barrier.^18^ The dietitians addressed this by providing simple recipes and easy-prep tips or semi-prepped grocery items to facilitate uptake (see cases 3, 4, and 5 in **Supplement Material ST1** for examples).

##### Domain III: Social and Family Influences

Caregiving responsibilities (e.g., children or parents) can reduce time for self-care, including meal preparation, which can be a significant barrier to adopting a healthy diet or maintaining a healthy lifestyle.^19^ There can also be peer pressure or social influences when adopting a new diet.^20^ To address this barrier, dietitians tailored their recommendations to involve the needs of the family members at home. For example, dietitians included family members in the food preparation and grocery selection and provided flexible scheduling (see practice case 3 in **Supplement Material SM1**).

##### Domain IV: Individual Beliefs and Knowledge

Individual beliefs and knowledge can act as barriers to adopting the DASH plan due to misinformation, health perceptions, fear of change, and knowledge gaps (see case 4 in **Supplement Material SM1**). A limited understanding of food’s impact on health can provide limited motivation to adjust eating patterns.

Individual beliefs and knowledge were addressed with motivational interviewing (MI) techniques, specifically prioritizing patient autonomy and decision making. Dietitians used the *‘elicit-provide-elicit’* MI strategy to give the participant control over what suggestions were offered to them.^21^ This strategy ensured their beliefs were incorporated into the counseling but also gave the dietitian the opportunity to provide other perspectives or information. The didactic modules were used to explain ‘why’ and more important, ‘how’ the DASH plan impacts their health.

##### Domain V: Culture Adaptation of DASH

The erasure of culture when discussing diet can alienate participants and in turn decrease the likelihood of diet adoption. As reported elsewhere, culture is a social determinant of health thus dietary guidelines and recommendations need to consider cultural personalization.^22^ Adapting the DASH plan to the participant’s style of eating was a major part of ‘the gate to plate’ model. During the first call, the dietitian gathered information on the participant’s eating habits and lifestyle, including cooking patterns and food preferences. The dietitian used this information when making food choice suggestions or referenced the cookbook when appropriate. The grocery order sheet was customized to each participant to include frequently ordered DASH-appropriate foods in order to promote autonomy in ordering and respect individual food preferences. By sending groceries instead of meals, participants could order the grocery components that matched their preferences within each DASH category. Lastly, through education modules, recipe adaption strategies were discussed like ingredient swaps for more DASH aligned cuisine components and DASH additions vs. removal of an ingredient were facilitated.

Table 2 highlights the DASH principles applied to four cuisines: soul food, Afro-Latin, African Heritage, and Afro-Caribbean. It also highlights high potassium (>250 mg/serving) foods in each of these cuisines with an asterisk. It is important to note that there is extensive diversity even within regional cuisine, so it is best to let the individual steer the decision making.

### Self-Reported Adherence and Promoting Engagement

During weekly calls, participants reported the number of meals and snacks consumed from non-study sources the prior week. These two metrics were used to determine each participant’s intervention adherence score via the following formula:

(1) $$\left(\frac{\left(\#\;\text{of typical meals each day}\cdot 7)-(\#\;\text{of meals outside groceries in a week}\right)}{\left(\#\;\text{of typical meals each day}\cdot 7\right)}\right)\cdot 100=\text{percent of meals the participant consumed from GoFresh that week}$$

The same formula was used for the percentage of snacks. Notably, this formula only focused on grocery adherence, which may not reflect DASH adherence. While outside foods were discouraged during the study, they could be DASH-compliant using the modules and knowledge gained for informed food decision making.

Leftover food from previous orders was also used to assess adherence. If there were many fruits and vegetables left over, then the dietitian dedicated additional counseling time on strategies to increase fruit and vegetable consumption. Adherence data will be published in future papers after the study has completed. Other objective measures of adherence are outlined in **Supplement Table ST3**.

## Conclusion

Prior trials demonstrated the ability of a low sodium DASH dietary pattern to improve CVD risk factors, with greater effects among Black adults.^3,4^ However, there is still a need to address barriers that limit adoption of this healthy dietary pattern in real-world settings. The GoFresh, dietitian-assisted, DASH-patterned grocery intervention allowed for the implementation of DASH in a personalized manner that aimed to address barriers related to accessing DASH groceries and potential gaps in knowledge and skills related to preparing DASH meals. Future work should continue to delineate and address the barriers related to choosing, obtaining, preparing, consuming, and maintaining the DASH plan through grocery selection across the globe.

## Supplementary Material

Supplementary Files

This is a list of supplementary files associated with this preprint. Click to download.

- SupplementMaterial.docx

## Figures and Tables

**Table 1:** DASH Eating Plan—Number of Food Servings by Weekly Calorie Level

Food Group	8400	9800	11200	12600	14000	16000	18200	21700
Grains	28–35	35–42	42	42	42–56	56–70	70–77	84–91
Vegetables	21–28	21–28	21–28	28–35	28–35	30–37	35–42	42
Fruits	21–28	21–28	21–28	28–35	28–35	30–37	35–42	42
Fat-free or low-fat dairy products	14–21	14–21	14–21	14–21	14–21	21	21	21–28
Lean meats, poultry, and fish	21 or less	21–28 or less	21–28 or less	42 or less	42 or less	42 or less	42 or less	42–63 or less
Nuts, seeds, legumes	3 per week	3 per week	3–4 per week	4 per week	4–5 per week	6 per week	7 per week	7 per week
Fats and oils	7	7	14	14–21	14–21	21	21	28

**Table 2:** DASH Foods Categorized by Cuisine

DASH Food Group	Soul Food	Latin Heritage	African Heritage	Afro-Caribbean
Fruits	Peaches, apples, bananas*, rhubarb*, strawberries, oranges, watermelon, cantaloupe*, honeydew, persimmons	Papaya, mango, oranges, avocado*, breadfruit*, star fruit, passion fruit*, melons*, guanabana*, guava*, pineapple, sapote*, bananas*, custard apple*, prickly pear	Bananas*, dates*, dried figs*, figs, grapefruit, honeydew, cantaloupe*, lemons, limes, mangos, oranges, papaya, pomegranates, pumpkin puree, tamarind, watermelon	Akee, avocados*, bananas*, dates*, dried figs*, figs, grapefruit, guava*, lemons, limes, mangos, honeydew, cantaloupe*, oranges, papaya, pomegranate, pumpkin puree, tamarind pulp*, watermelon
Vegetables	Collard greens, bell peppers, onions, acorn squash*, yellow squash, zucchini, turnips, turnip greens, beets, beet greens*, okra*, potatoes*, sweet potatoes*, corn, cucumber, tomatoes, mustard greens*	Tomatoes, onions, peppers (both sweet and hot), yuca*, batata*, plantains* (both green and ripe), potatoes*, summer squash, pumpkin, chayote, heart of palm, spinach*, collard greens, cabbage*, carrots*, name*, tomatillos, corn, yams*	Asparagus, beets*, Brussels sprouts*, broccoli*, butternut squash*, red cabbage*, green cabbage, carrots*, eggplant, okra*, onions, bell peppers, radish*, scallions*, acorn squash*, yellow squash, zucchini, jicama, callaloo*, chard*, collard greens, kale*, mustard greens*, plantains*, spinach*, turnip, tomatoes and canned tomato varieties (no salt added), potatoes*, sweet potatoes*, yams*, yuca*	Broccoli*, butternut squash*, red cabbage*, green cabbage, carrots*, eggplant, okra*, onions, bell peppers, scallions*, acorn squash*, yellow squash, zucchini, callaloo*, chard*, collard greens, kale*, mustard greens*, plantains*, spinach*, turnip, tomatoes and canned tomato varieties (no salt added)
Grains	Grits, cornbread, rice, cornmeal, sorghum, millet, wheat breads, pasta	Maize (corn), rice, tortillas (flour and corn), pasta, bread, barley, cracked wheat	Amaranth, barley, couscous, maize/corn, rice varieties, sorghum, teff, wild rice, oats	Barley, couscous, maize/corn, oats, rice varieties, wild rice
Lean proteins	Chicken*, pork*, catfish, shrimp, oysters, crawfish*, turkey, beef*, crab*	Chicken*, beef*, pork*, goat*, cod*, salmon*, tuna*, clams*, mussels*, octopus*, sea bass*, shrimp, scallops, squid	Chicken*, turkey, eggs, lean beef*, lean pork*, goat*, cod*, haddock*, salmon*, halibut*, shrimp, scallops, canned tuna, canned salmon*, red snapper	Chicken*, turkey, eggs, lean beef*, lean pork*, goat*, cod*, haddock*, salmon*, halibut*, shrimp, scallops, canned tuna, canned salmon*, red snapper
Dairy	Milk*, cheeses	Fresh cheese (queso blanco), milk*, crema, yogurt	Coconut milk (light), Yogurt, Almond milk, Soy Milk	Coconut milk, light
Nuts, seeds, and legumes	Black eyed peas, red beans*, lima beans*, peanuts*, sesame seeds, cowpeas, pecans*	Black beans*, red beans*, lentils*, peanuts*, pigeon peas (gandules)*, coconut*, almonds*, cashews*, pumpkin seeds*	Black-eyed peas, butter beans, chickpeas, kidney beans*, lentils*, lima beans*, pigeon peas*, Brazil nuts*, cashews*, coconut*, peanuts*, pecans*, pumpkin seeds*, sunflower seeds*	Black-eyed peas, butter beans, chickpeas, kidney beans*, lentils*, lima beans*, pigeon peas*, peas, cashews*, coconut*, peanuts*, pumpkin seeds*
Spices and seasonings	Hot chiles, vinegar, garlic, molasses, file powder, paprika, onion powder, garlic powder, oregano, thyme, chicken broth, cinnamon, nutmeg, allspice, ginger	Hot chiles, achiote, cilantro, epazote, cumin, oregano, chili powder, cilantro, thyme, ginger, garlic, bitter orange, lime	Hot peppers and chilies, no salt added and low-sodium broths, vinegars, bay leaf, cinnamon, cilantro, cloves, coriander, cumin, curry, dill, garlic powder, ginger, mustard, nutmeg, onion powder, oregano, paprika, parsley, peppers, sage, sesame	Hot peppers and chilies, no salt added and low-sodium broths, vinegars, bay leaf, cinnamon, cilantro, cloves, coriander, cumin, curry, dill, garlic powder, ginger, mustard, nutmeg, onion powder, oregano, paprika, parsley, peppers, sage, sesame, turmeric

* High potassium foods (>250 mg per 100 g)

## Data Availability

Data sharing is not applicable to this article as no datasets were generated or analysed during the current study.

## Abbreviations

DASH: Dietary Approaches to Stop Hypertension
GO: weekly grocery ordering call
GO#: week number of 12-week intervention
RZ: randomization visit
FV1: follow up visit 1 (3-month mark)
FV2: follow up visit 2 (6-month mark)
FV3: follow up visit 3 (12-month mark)
MI: motivational interviewing
